# Allosteric Activation of the ATPase Activity of the *Escherichia coli* RhlB RNA Helicase*

**DOI:** 10.1074/jbc.M708620200

**Published:** 2007-12-28

**Authors:** Jonathan A. R. Worrall, Françoise S. Howe, Adam R. McKay, Carol V. Robinson, Ben F. Luisi

**Affiliations:** ‡ Department of Biochemistry, University of Cambridge, 80 Tennis Court Road, Cambridge CB2 1GA; § Department of Chemistry, University of Cambridge, Lensfield Road, Cambridge CB2 1EW, United Kingdom

## Abstract

Helicase B (RhlB) is one of the five DEAD box RNA-depend-ent ATPases found in *Escherichia coli*. Unique among these enzymes, RhlB requires an interaction with the partner protein RNase E for appreciable ATPase and RNA unwinding activities. To explore the basis for this activating effect, we have generated a di-cistronic vector that overexpresses a complex comprising RhlB and its recognition site within RNase E, corresponding to residues 696–762. Complex formation has been characterized by isothermal titration calorimetry, revealing an avid, enthalpy-favored interaction between the helicase and RNase E-(696–762) with an equilibrium binding constant (*K_a_*) of at least 1 × 10^8^ M^-1^. We studied ATPase activity of mutants with substitutions within the ATP binding pocket of RhlB and on the putative interaction surface that mediates recognition of RNase E. For comparisons, corresponding mutations were prepared in two other *E. coli* DEAD box ATPases, RhlE and SrmB. Strikingly, substitutions at a phenylalanine near the Q-motif found in DEAD box proteins boosts the ATPase activity of RhlB in the absence of RNA, but completely inhibits it in its presence. The data support the proposal that the protein-protein and RNA-binding surfaces both communicate allosterically with the ATPase catalytic center. We conjecture that this communication may govern the mechanical power and efficiency of the helicases, and is tuned in individual helicases in accordance with cellular function.

RNA helicases are a diverse set of proteins found in all three kingdoms of life that possess the ability to unwind short stretches of RNA duplexes in reactions that require the hydrolysis of nucleoside triphosphates (1). They are the largest group of enzymes in eukaryotic RNA metabolism and are involved in virtually all aspects of cellular RNA manipulation including transcription, splicing, RNA nuclear export, and ribosome biogenesis (2, 3). The DEAD box helicases are grouped in helicase superfamily 2, whose members contain nine conserved motifs, including the Walker B motif DEAD from which they take their common name (Fig. 1). *Escherichia coli* contains five genes encoding for DEAD box proteins; *csdA* (formerly called *deaD*), *dbpA*, *rhlB*, *rhlE*, and *srmB* (4). The gene products have a common core of 350–400 amino acids, consisting of two domains that have the same fold found in the DNA-recombination protein RecA. Flanking this two-domain core are variable regions that are thought to be responsible for the differing properties and functions of the five helicases (4).

DEAD box helicases can unwind short RNA duplexes that are no more than two helical turns in length (~22 bp), and some helicases can displace avidly bound proteins from RNA (1,5). A role of DEAD box RNA helicases as nonspecific RNA unfolding enzymes has been described (6). Yang and Jankowsky (7) have elegantly shown that DEAD box helicases use a single strand RNA region to facilitate loading onto duplex RNA where only a few base pairs are disrupted in an ATP-dependent manner, leading to destabilization of the remainder of the duplex and its spontaneous disassembly. Duplex unwinding by the RNA helicases differs from that of DNA or viral helicases, which in contrast act as highly processive translocases that may peel off many hundreds of complementary RNA and DNA strands in their paths (5).

The crystal structure of the *Drosophila* DEAD box helicase Vasa in complex with a non-hydrolyzable ATP analogue and single-stranded RNA lends support to the proposed mechanism of duplex unwinding by the DEAD box family (8). This complex structure shows that ATP binding promotes relative movement of the two RecA-like domains to induce a closed form of the helicase. The adoption of the closed conformation organizes the catalytic site and so enables hydrolysis of the terminal phosphate group of the ATP. The marked bending of the single-stranded RNA is suggestive of a mechanism that could physically disrupt a few RNA basepairs in a duplex (5, 8).

RNA helicases are often part of large macromolecular assemblies, and it is becoming increasingly apparent that their ATPase and/or RNA helicase activities can be modulated by the interactions that are formed within these complexes (3, 9 –12). One such example is the exon-junction complex, in which the ATPase activity of the DEAD box helicase component (eIF4AIII) is impeded by interaction with RNA and partner regulatory proteins (13,14). Turning to examples of bacterial helicase complexes, the *E. coli* DEAD box ATPase RhlB forms part of the multiprotein assembly known as the RNA degradosome. This large complex also comprises the endoribonuclease, RNase E, the exoribonuclease, polynucleotide phosphorylase, and the glycolytic enzyme, enolase. The co-localization of these enzymes in the degradosome allows cooperation of their activities (15, 16). The presence of RhlB in the degrado-some has been shown to facilitate RNA degradation by RNase E (17) and polynucleotide phosphorylase (18–20). This facilitation is presumably due to the removal of secondary structure from the substrate RNA (20).

RhlB interacts with the non-catalytic C-terminal “scaffold” domain of RNase E, which has also been shown to be required for polynucleotide phosphorylase and enolase binding (21). The C-terminal domain is predicted to be predominantly unstructured, with some small regions of propensity for secondary structure being restricted to specific sites of recognition (22) . The site of interaction on RNase E for RhlB has been identified to lie between residues 698 and 762 (23). This engages the C-terminal RecA-like domain of the helicase (24), and the binding surface has been proposed to encompass residues 368 to 397, which are not part of a conserved helicase signature motif (23) . From a homology model of RhlB based on the *Drosophila* Vasa structure this binding site is at a comparatively large distance of around 20 Å from the ATP binding pocket (Fig. 1). Consequently, the binding of RNase E must have an indirect effect on the ATPase activity of RhlB. Predictive methods have identified an exposed coil between residues 377 and 383 (Fig. 1) as a putative binding site (23). This site is similar to the surface used in the eIF4AIII DEAD box helicase, with its partner proteins in the exon junction complex (13, 14).

RhlB is the only *E. coli* DEAD box protein that requires a protein partner to stimulate its ATPase activity (10,25,26). The stimulating effect of RNase E on RhlB does not require the RNA-binding sites of the ribonuclease that flank the helicase interaction site (18), suggesting that the boost of ATPase activity is not caused indirectly by increased recruitment of RNA. Indeed, we observe that the RNase E/RhlB interaction actually decreases the RNA affinity of the helicase.^4^ Instead, the activation is likely to involve induced conformational changes in the RhlB, but the nature of these changes is not presently clear. Chandran *et al*. (23) have identified a distinctive sequence difference in one of the nine conserved motifs that may account for the activation of RhlB. The terminal amino acid of motif V is an Asp in all *E. coli* DEAD box proteins but is replaced in RhlB with a His (Fig. 1). Notably, residues in the predicted binding region for RNase E are co-conserved together with the motif V His residue, suggesting a link between RNase E binding on the one hand and the activation of ATPase activity on the other (23). This link is explored as part of the present work. Our results confirm that there is communication between the ATPase catalytic site and the putative RNase E binding site; however, they also indicate that the activating effect of RNase E binding on ATPase activity cannot be ascribed to a few surface and active-site residues. Instead, the communication requires a constellation of amino acids that collectively affect the active site pocket.

In the course of designing an accommodating cavity in the ATP binding pocket, we inadvertently discovered that substitution of residue Phe-10 near the adenine ring boosted ATPase activity tremendously. However, no activity could be detected in the presence of RNA, indicating that RNA binding has locked the enzyme into an inactive state. We discuss the implications for understanding how the enzyme might transduce energy from ATP hydrolysis into mechanical work.

## Experimental Procedures

### RhlB Expression and Site-directed Mutagenesis

Expression systems for three *E. coli* RNA helicases, RhlB, RhlE, and SrmB, were provided by A. J. Carpousis, CNRS Toulouse, France. The recombinant helicase genes are inserted within the multiple cloning site of a Novagen pET11a (Amp^r^) vector under the control of the T7 promoter. Mutations in each of the recombinant genes were created using a procedure based on the Stratagene QuikChange mutagenesis kit (27). The forward and reverse primers used to introduce the respective mutations in the RNA helicases are listed in Table 1. All clones were sequenced to corroborate that the intended mutations were successfully introduced. Site-directed variants of RhlB were also constructed in the di-cistronic pRneRhlB vector (see below) using the mutagenic primers reported in Table 1. Expression and purification was the same as outlined above for the wild-type complex.

### Recombinant Expression and Purification of RNA Helicases and Variants

Wild-type (WT)^5^ and site-directed variants of RhlB, RhlE, and SrmB were expressed in *E. coli* strain BL21(DE3) and isolated and purified as previously described (22,23). A final purification step was introduced to the published procedure involving S200 size exclusion chromatography.

### Construction and Expression of the RNase E-(696 –762)/RhlB Co-expression Vector

The Novagen pRSF-Duet-1 (Kan^r^) di-cistronic vector was used to construct a co-expression system for the complex formed between RhlB and residues 696 –762 of RNase E. The vector consists of two multiple cloning sites each under the control of a T7 promoter. Primers for polymerase chain reaction (PCR) amplification were designed with restriction enzymes sites added for facile cloning of the products into the multiple cloning sites of the pRSF vector. The *rhlB* gene was amplified form the pET11a vector and ligated in the upstream NdeI and downstream XhoI sites in the second pRSF multiple cloning site. DNA encoding for amino acid residues 696 –762 of RNase E was amplified from the full-length RNase E in a pET15b vector. The product was cloned in the upstream BamHI and downstream SalI sites of the first pRSF multiple cloning site creating a His_6_ tag at the N terminus. Several clones were evaluated by restriction digests and sequencing in both multiple cloning sites to corroborate the correct insertion of the amplified genes into the vector. The final vector was named pRneRhlB. A C-terminal truncation of RhlB was constructed in the pRneRhlB vector. The codon (CTC) for amino acid 398 (Leu) in RhlB was replaced by a stop codon (TAA) using the QuikChange strategy. The final clone was designated pRneRhlBΔ1–397.

*E. coli* BL21(DE3) cells were transformed with pRneRhlB or pRneRhlBΔ1–397 and single transformants were transferred to 50 ml of 2× YT medium containing 50 *μg* liter ^1^ kan and incubated overnight at 37 °C. Two-liter Erlenmeyer flasks containing 500 ml of 2× YT medium and 50 *μ*g liter ^1^ kan were inoculated with 5 ml of overnight culture and incubated at 37 °C until an *A*_600_ of 0.5– 0.6 was reached. At this point expression of the recombinant genes was induced with 1 mM isopropyl *β*-D>-thiogalactopyranoside. After 3 h the cells were harvested and resuspended in lysis buffer (50 mM Tris/HCl, pH 8.0, 200 mM NaCl, 100 mM KCl, 5 mM MgCl_2_, 5 mM imidazole, and an EDTA-free protease inhibitor mixture tablet) and passed three times through an Emulsiflex-05 cell disruptor (Avestin). The soluble fraction was collected by centrifugation (35,000 × *g*, 4 °C) and loaded to a Ni-NTA Hitrap column (GE Healthcare). Extensive washing with lysis buffer was followed by a gradient elution with lysis buffer supplemented with 500 mM imidazole (buffer B). Two peaks eluted at 20 and 40% buffer B and frac-tions were analyzed by SDS-PAGE electrophoresis. The first peak contained RhlB and the second peak was enriched with RhlB and the His_6_-tagged RNase E-(696–762). Fractions from the second peak were pooled and dialyzed against 50 mM Tris/ HCl, pH 8.0, and 50 mM NaCl (buffer C) and loaded to an SP column (GE Healthcare) equilibrated in buffer C. A linear gradient (0 –100% buffer C containing 1 M NaCl) was applied and a major peak eluted at ~350 mM NaCl. Fractions were pooled, concentrated in 30-kDa cut-off Centricon units (Vivascience), and loaded to an S200 size exclusion column (GE Healthcare). A major peak eluting at a column volume corresponding to a species with a molecular mass of ~50 kDa was obtained. Analysis of this peak by SDS-PAGE electrophoresis revealed it to contain the complex between RhlB and RNase E-(696–762).

### Construction and Purification of RNase E-(696 –762) Peptide

A plasmid with the DNA sequence for overexpression of RNase E residues 696 –762 was created. The PCR-amplified DNA fragment of RNase E-(696–762) was ligated into the upstream BamHI and downstream SalI sites in the first multiple cloning site of the pRSF-Duet-1 vector. Overexpression in 2× YT medium supplemented with 50 *μ*g liter ^–1^ yielded a protein product with an N-terminal His_6_ tag that eluted from a Ni-NTA column as a broad peak. Fractions analyzed by SDS-PAGE electrophoresis showed a major band running at ~18 kDa. These fractions were pooled, concentrated, and applied to an S200 size exclusion column.

### Isothermal Titration Calorimetry

Protein samples for isothermal titration calorimetry (ITC) analysis were dialyzed extensively against 50 mM potassium phosphate, pH 7.4, 100 mM NaCl, and 1 mM dithiothreitol for 24 h at 4 °C. After dialysis samples were concentrated using either 30- or 5-kDa cut-off Centricon units (Vivascience) and concentrations were determined from UV spectroscopy using extinction coefficients (*∊*) at 280 nm of 36,120 M ^-1^ cm ^-1^ for RhlB and variants, and 1,490 M ^1^ cm for RNase E-(696–762). RNase E-(696–762) solutions of 300 *μ*M were placed into the syringe and titrated into the sample cell containing 20 *μ*M WT RhlB or a site-directed variant with stirring at 310 rpm during the experiment. All titration experiments were performed at 25 ± 0.1 °C on a Microcal VP-ITC calorimeter with an injection volume of 2 *μ*l for the first and 5 *μ*l for all subsequent titration points, 60-s initial equilibrium delay and 270-s pause between injections. Binding isotherms were analyzed with Microcal Origin 7.0. In each case the first data point was discarded and the baseline adjusted manually. The integrated data were corrected for the heat of dilution of RNase E-(696 –762) into the buffer and binding isotherms were analyzed with 1:1 and 2:1 binding models using the software package of the manufacturer (28).

### Nanospray Mass Spectrometry

The “apo”-RhlB (in the absence of the RNase E peptide) and the co-expressed complex, after size exclusion chromatography, were prepared to concentrations between 5 and 20 *μ*M and buffer exchanged into 250 mM ammonium acetate (Micro Bio-Spin chromatography columns, Bio-Rad). All spectra were acquired on a Q-ToF 2 modified for high mass operation and equipped with a Z-spray nanoflow source (Waters, Manchester, UK) (29). The following experimental parameters were used: capillary voltage 1.7 kV, cone voltage 80–120 V, cone gas 100 liter h^-1^ collision cellvoltage up to 200 V, ion transfer stage pressure 4.0 × 10 ^-3^ to 2.0 × 10 ^-2^ mbar, argon collision gas at a collision cell pressure of 2–7 *μ*bar. External calibration was achieved by using a 33 mg ml^–1^ aqueous solution of cesium iodide (Sigma). Calibration, acquisition, and processing were carried out using MassLynx software (Waters, Manchester, UK).

### ATPase Assays

ATPase activity of RNA helicases was monitored spectrophotometrically on a Shimadzu BioSpec-1601 UV-visible spectrophotometer thermostatted at 25 ± 0.1 °C using the Molecular Probes EnzCheck Phosphate Assay kit (Invitrogen). The assay is based on a method originally described by Webb (30). In the presence of inorganic phosphate (P_i_) released by hydrolysis of ATP, the substrate 2-amino-6- mercapto-7-methylpurine riboside is converted by purine nucleoside phosphorylase (PNP) to ribose-1-phosphate and 2-amino-6-mercapto-7-methylpurine. This enzymatic conversion is accompanied by a shift in the maximum absorbance from 330 nm for the substrate to 360 nm for the product. Assays were performed in 1-ml volumes in 50 mM Tris/HCl, pH 7.5, and supplemented with 200 *μ*M ATP and 400 *μ*M MgCl_2_. Bakers’ yeast RNA (Sigma), when included, was at a final concentration of 40 *μ*g/ml. Reaction components were preincubated for 10 min at room temperature before the reaction was started by the addition of the desired helicase or helicase-RNase E complex to a final concentration of 2.4 *μ*M. Reactions were monitored for 300 s with initial rates calculated from the rate curve over the first 30 s converted into the amount of phosphate released/min/mol of protein from a P_i_ standard curve. Reported rates are an average of two or three independent experiments.

## Results

### Site-directed Mutagenesis of Three E. coli DEAD Box Proteins and the Co-expression of an RNase E·RhlB Complex

Site-directed variants of three *E. coli* RNA DEAD Box helicases, RhlB, RhlE, and SrmB were constructed (Table 1). The final expression constructs produced proteins that were highly overexpressed in *E. coli*, were entirely soluble, and could be purified to >95% as judged from SDS-PAGE. From previous studies it was found that RhlB forms a complex with RNase E (19,21–23). To aid in investigating the properties of this complex, a di-cistronic expression vector was constructed encoding WT RhlB or its site-directed variants and an N-terminal His_6_- tagged 67-amino acid peptide of RNase E corresponding to residues 696–762. Elution from a Ni-NTA column followed by S200 size exclusion chromatography revealed the RNase E peptide to remain bound to RhlB, indicating that the interaction is avid (Fig. 2). The normalized intensities of the bands on a denaturing gel suggest a 1:1 stoichiometry, corrected for dye uptake in proportion to the molecular mass. It is noted that the Coomassie-stained band for the RNase E peptide runs at twice the apparent molecular mass (~18 kDa) than the predicted 9163 Da, which includes the 67 amino acids corresponding to RNase E residues 696–762 and 13 additional amino acids coming from the plasmid of which six are the N-terminal His tag. Non-dissociative mass spectrometry analysis provides a stoichiometry of 1:1 and a molecu-lar mass of the RNase E peptide of 9189 Da (Fig. 2). The binding of one RNase E to one RhlB is in accord with earlier results using the R-domain construct of RNase E (residues 628 – 843), which includes the two putative RNA binding domains flanking the RhlB binding site (Fig. 2) (22, 23).

### Analysis of Interactions of RhlB Surface Mutants with RNase E

Residues Ser-381 and Tyr-383 of RhlB were predicted previously to be important for binding/recogni- tion of RNase E (23). To test this, the S381A and Y383A mutants of RhlB were overexpressed and purified. An expression plasmid to overexpress N-terminal His_6_-tagged RNase E, residues 696–762, was also constructed. The peptide was analyzed by SDS-PAGE and it migrates with the same apparent size as seen for the co-expressed RhlB·RNase E-(696–762) complex (data not shown).

Representative ITC titrations for the binding of RNase E-(696–762)

to WT, and RhlB variants are shown in Fig. 3, and the derived thermodynamic parameters of all the complexes investigated summarized in Table 2. All data have been corrected for the heat of dilution of RNase E-(696–762), estimated from titrating RNase E-(696–762) into the buffer solution. For all complexes for which binding was detected the interaction is exothermic (Fig. 3) and driven by a highly favorable enthalpy change. The data were fit best to a simple binary interaction model with the stoichiometry of binding close to unity (Table 2), confirming the non-dissociating mass spectrometry data for the co-expressed WT complex.

The S381A variant has significantly smaller heat release compared with WT, which may indicate a change in hydrogen bonding interactions with the RNase E peptide. Because the binding is strong, only a lower limit can be placed on the association constant, and it is not possible to evaluate if this differs from the WT.

No detectable heat change was observed for the titration of RNase E-(696–762) into the Y383A variant of RhlB in the ITC experiment. This would seemingly indicate that removal of Tyr-383 abolishes the ability of RhlB to bind RNase E. However, from size exclusion data it was found that the Y383A variant elutes very close to the void volume of the column, suggesting the mutation causes the formation of a higher order molecular weight species (Fig. 4). This contrasts with the behavior of WT RhlB and all other helicases used in the present work, which elute later and are likely to be monomers based on our current and earlier mass spectrometry data (22).

### ATPase Activity of Three E. coli DEAD Box Helicases

ATPase activities of RhlB, RhlE, and SrmB were measured using a purine nucleoside phosphorylase colorimetric assay (30). Representative rate curves generated from the assay with the different helicases are shown in Fig. 5 and the data are summarized in Table 3. The reactions were done at ATP concentrations exceeding the expected *K_m_*. The ATPase activity of RhlB is the lowest of the three studied *E. coli* helicases, which agrees with previous findings (20). A slight stimulation of RhlB activity in the presence of *Saccharomyces cerevisiae* bulk RNA was observed, although this was again at the lower detection limit of the assay. RhlE and SrmB were clearly stimulated in the presence of RNA; RhlE activity increased 11-fold and SrmB by 4-fold. Earlier studies found that the activity of RhlE is greater than SrmB for many different *E. coli* RNA (25). We observe similar rates for both SrmB and RhlE, but this may be due to the nature of the RNA substrate (*S. cerevisiae* bulk RNA). The coexpressed complex, RhlB·RNase E-(696–762), showed negligible activity in the absence of RNA. Similar ATPase activity was seen for longer constructs of RNase E-(628–843), suggesting that the smaller fragment encompassing the binding site (696–762) is sufficient for the weak stimulatory effect (data not shown). The ATPase activity was stimulated in the presence of RNA (Fig. 5 and Table 3). Nevertheless, the activity is still some 7 times lower than RhlE and SrmB in the presence of RNA. Together, these data corroborate that RhlB is a “weak” ATPase even in the presence of its protein activator RNase E (19).

The basic C-terminal tail of RhlB containing residues 397– 421 is thought to interact with RNA, because RhlB lacking the C-termi-nal tail binds RNA more weakly than the full-length RhlB (23). The ATPase activity for the co-expressed RhlB(Δ1–397)·RNase E-(696–762) complex was not enhanced in the presence of RNA relative to the full-length RhlB·RNase E complex (Table 3), confirming the involvement of the C-terminal tail in recruiting RNA to the helicase. Similar results have been seen for the DEAD box heli-cases DdpA (31) and the eukaryotic DEAD box helicase CYT-19 (32).

### The Effect of the Terminal Residue in Motif V on ATPase Activity

**Figure 1:**
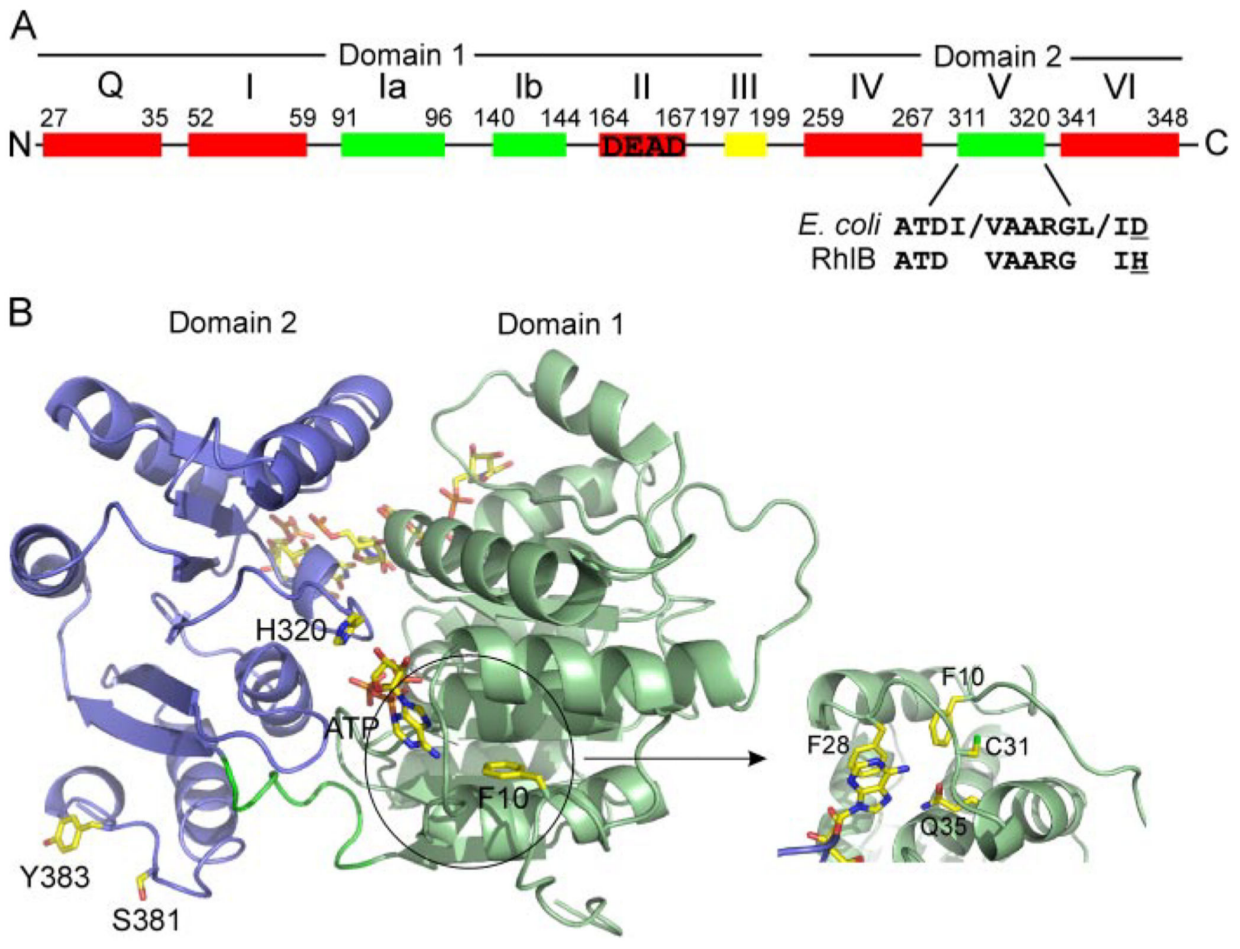
*A*, location of the nine conserved sequence motifs in the DEAD box helicase RhlB. Color scheme indicates motifs involved in ATP binding and hydrolysis (*red*), RNA binding (*green*), and ATP-induced conformational change (*yellow*). Motif II is the Walker B motif, DEAD. The consensus amino acid sequence of motif V for *E. coli* DEAD box helicases is shown together with the sequence of RhlB highlighting the change in the final amino acid (*underlined*). *B*, homology model of RhlB, with domains 1 and 2 colored *green* and *blue*, respectively. The ATP, RNA, and locations of the residues mutated in this study are shown in stick representation. The *inset* is a close-up of the helix-loop-helix that forms theQ-motif in DEAD box helicases, with Phe-28 stacking with the adenine base and the invariantly conserved Gln-35 residue positioned to form hydrogen bonds with the N-6 and N-7 positions of the adenine base.

**Figure 2:**
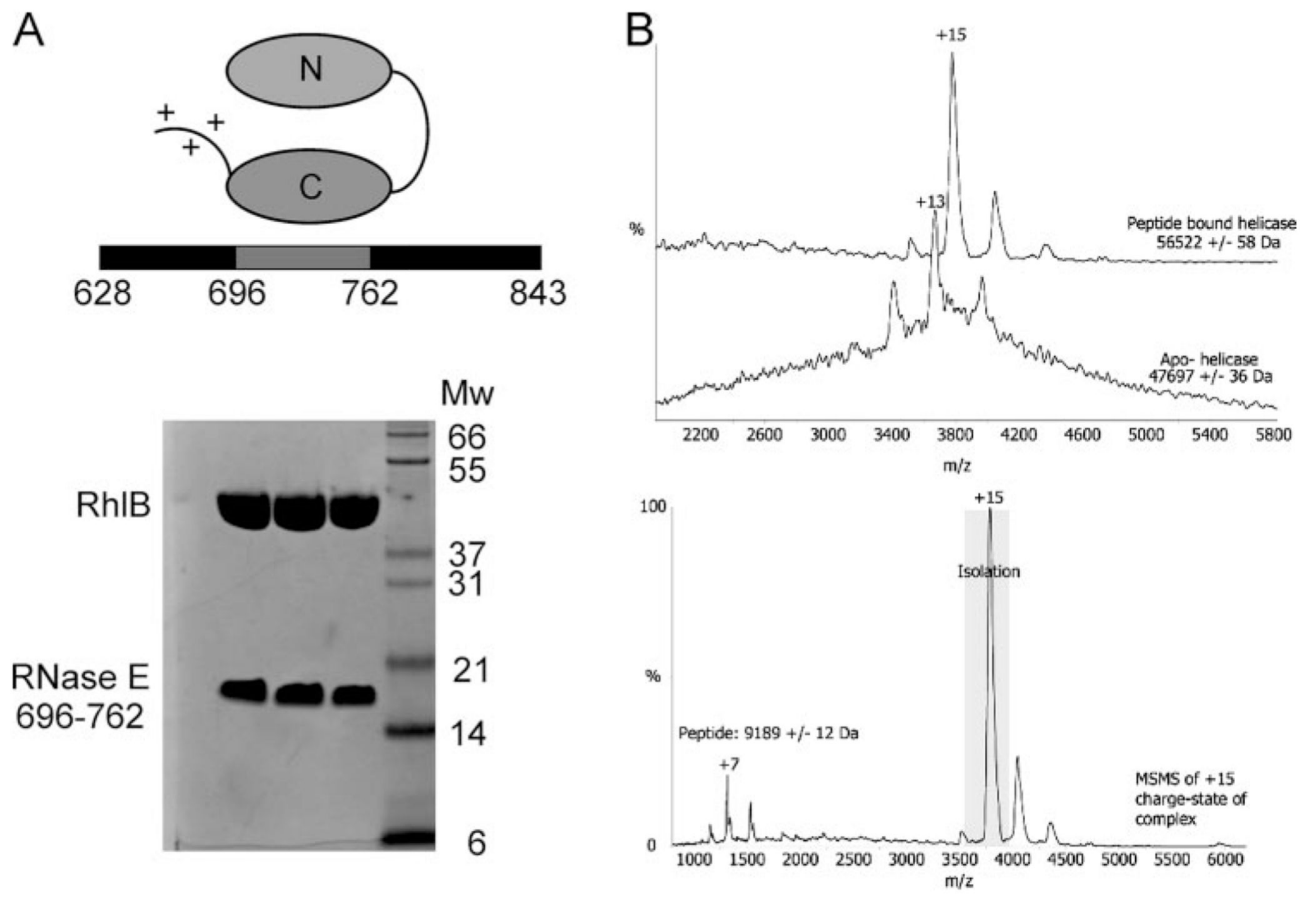
Co-expression of the complex between RNase E residues 696-762 and RhlB. *A*, schematic of the interaction between RhlB and RNase E. The C-terminal domain of RhlB interacts within a region of RNase E encompassing residues 696–762. Either side of this region are predicted RNA binding sites. Coomassie-stained bands on an SDS-PAGE gel of consecutive fractions after S200 size exclusion chromatography reveal a complex between the RNase E peptide and RhlB. *B*, mass spectrometry analysis. Nanoflow ESI-mass spectra (*top*) under non-dissociating conditions of apo-RhlB (in the absence of the RNase E peptide) and the co-expressed complex after size exclusion chromatography. MS/MS analysis of the +15 ion of the RNase E-(696–762)·RhlB complex. The +7 ion corresponds to the RNase E peptide dissociated from the RhlB.

Motif V is part of the RNA-binding motif in the *Drosophilia* Vasa crystal structure, but a role in ATPase activity or in coupling the ATPase and helicase activities has been indicated (33). From the homology model of RhlB, motif V is part of a loop at the interface between domains 1 and 2 that point toward the RNA binding region and is in contact with the nucleoside triphosphate (23). In *E. coli* four of the five DEAD box proteins have an Asp as the final residue of the motif. For RhlB this Asp is replaced by a His (Fig. 1). This difference has been proposed to account in part for the requirement of RhlB for a protein partner to stimulate its ATPase activity (23).

The helicase mutation H320D in RhlB was purified and tested for ATPase activity. For comparison, the effect of the converse substitution, from Asp to His, was studied in RhlE (D310H) and SrmB (D313H). These substitutions are effectively neutral in RhlE and SrmB, with comparable activity to WT both in the absence and presence of RNA (Table 3). For RhlE D310H, ATPase activity was marginally higher than for the WT protein, with a similar situation encountered for SrmB (Table 3). In striking contrast, the RhlB H320D variant had no detectable ATPase activity in either the absence or presence of RNA. To explore whether the mutant would become activated by interaction with RNase E, the RhlB (H320D)·RNase E-(696 – 762) complex was prepared using a di-cistronic expression vector. Despite the presence of RNase E-(696–762) the ATPase activity was still not detectable, either in the absence or presence of RNA. This variant complex behaved similarly to the WT complex on size exclusion chromatography, and ITC analysis shows it has similar binding stability compared with the WT, confirming that RNase E-(696–762) and RhlB (H320D) are forming an avid interaction in the inactive complex (Table 2). Circular dichroism spectra confirm that the protein has the same helical content and structural stability as the wild-type protein (data not shown). Thus, the lack of activity cannot be due to destabilization of the structure or lack of interaction with RNase E. Instead, it must arise from small structural changes in the catalytic site.

These results suggest that for RhlE and SrmB either an Asp or a His in motif V has little significance on the rate of ATP hydrolysis. However, for RhlB a clear preference for His over Asp in motif V is revealed. The impact of this residue is therefore highly dependent on the context of the other residues in the enzyme that will influence how the key catalytic residues are maneuvered during formation of the transition state.

### ATPase Activity of S381A and Y383A Variants of RhlB

To explore the possible communication of surface residues and ATPase catalytic site, ATPase activity of the S381A variant was assayed. In the absence of the RNase E peptide, S381A behaved like the WT RhlB. Upon addition of RNase E-(696–762) in stoichiometry and RNA, measurable ATPase activity was observed (Table 3). We also examined the ATPase activity of the Y383A variant, but no detectable activity was observed in the absence or presence of RNase E-(696–762) or RNA, which may be due to the oligomerization of this mutant.

### Evidence for Allosteric Communication between the RNA Binding Surface and the Catalytic Center

ATP is predicted to bind RhlB in a cleft at the interface of the two RecA-like domains. The adenine base is held in place through a stacking interaction with a conserved aromatic residue and hydrogen bonding interactions with a Gln side chain (Fig. 1*B*). These residues form part of the recently assigned Q-motif, so called because of the invariant Gln residue (34). The motif is part of a highly conserved substructure consisting of a helix-loop-helix and is found only in the DEAD box RNA helicases (34). We were interested in forming a small cavity in this pocket to accommodate the bulky substituent of an inhibitor. Based on the homology model we chose to replace Phe-10, which is conserved in the other *E. coli* DEAD box helicases, with Ala and Met. This aromatic residue is in physical proximity to the residues of the Q-motif and is proposed to contribute hydrophobic interactions with residues within the motif.

Purification of the Phe-10 variants initially proved problematic with low yields and high instability encountered. Cordin *et al*. (35) report similar problems for the equivalent substitution in the eukaryotic DEAD box, Ded1, which hampered their attempts to study this mutation *in vitro*. For the RhlB Phe-10 variants, these problems were overcome by co-expressing the variants with RNase E-(696–762). Both RhlB F10A and F10M exhibited an enhanced rate of ATP hydrolysis to such an extent that the activity of F10M/RNase E-(696–762) in the absence of RNA was at a similar level to WT RhlB/RNase E-(696–762) in the presence of RNA (compare data in Tables 3 and 4). However, inclusion of RNA abolished the hydrolysis of ATP by both F10M and F10A mutants (Table 4). These findings may indicate that the hydrolytic site cannot maneuver to form the transition state because the internal movements are impeded as a result of RNA binding.

## Discussion

Some RNA helicases require accessory proteins or co-factors to carry out their specific functions. Partners for these DEAD box proteins have been identified by genetic or physical interaction studies (36). However, the effects of only a very few cofactors or accessory proteins on the enzymatic functions of RNA helicases have been characterized *in vitro*. As part of the present work we have extended previous studies that have reported on the *E. coli* DEAD box RNA helicase, RhlB, whose ATPase activity is stimulated by interaction with its partner protein, the endoribonuclease RNase E (19, 22, 23). Evidence indicates that the boost of ATPase activity is not caused by increased recruitment of RNA (18). Notably in this regard, we observe that the RNase E/RhlB interaction decreases the RNA affinity of the helicase using a filter-binding assay^4^. Instead the activation of ATP turnover is likely to involve a conformational change. In this study, we have mutated residues that were proposed to lie either on the RNase E-engaging surface or to communicate between that surface and the active site.

An earlier proposal suggested that a His residue in motif V of RhlB is important for communicating the stimulatory effect of RNase E binding to the ATPase catalytic site (23). In homologues of related DEAD box proteins from bacteria and many eukaryotic proteins, this motif V is an Asp rather than a His. From the crystal structure of Vasa, motif V is seen to be the only motif that interacts with both the RNA binding region and nucleoside triphosphate, with the carboxyl group of Asp-554 (equivalent to His-320 in RhlB) forming a H-bond with the 3’-OH group of the ribose (8). The importance of this interaction in Vasa is demonstrated by the loss of 50% of the ATPase activity by the substitution of Asp-554 to Ala (8). Furthermore, in the DEAD box protein Prp28, mutations of the corresponding Asp in motif V have a detrimental effect on yeast growth (37). Mutations in the DE*X*H box splicing factor Prp22 have shown a direct role in stimulating ATPase activity upon RNA binding (38).

We had originally thought that single substitution of His to Asp in motif V might boost the ATPase activity of the RhlB so that it became as active as its *E. coli* paralogues, SrmB and RhlE. In the homology model of RhlB, the His-320 side chain is orientated in such a way that a polar interaction of the imidazole side chain with the ribose sugar is favored (Fig. 1). Our results show that the effective substitution of one H-bonding residue for another in SrmB or RhlE has no effect on ATPase activity, whereas for RhlB the substitution completely inhibited the activity regardless of whether RNA or RNase E-(696–762) was present (Table 3). It therefore seems that the mechanism of activation by RNase E in RhlB requires participation of other residues in addition to the motif V His or Asp.

The helicases generally have large *K_m_* values for ATP with values ranging from a little less than 0.1 to 100 *μ*M (39–41). In accord with those measurements, we did not detect any heat change by ITC for the interaction of WT RhlB or its RNase E-(696 –762) complex with ATP or the non-hydrolyzable ATP analogue, ADP-PNP (data not shown). Like the WT enzyme, the inactive H320D mutant also gave no measurable heat change with ATP or ADP-PNP in either its free or RNase E-(696 –762) bound forms. It is unlikely that the binding of the nucleotide is purely entropic, and some enthalpy change is expected. Thus, the lack of heat release indicates poor affinity for the substrate, as might be expected for a classical enzyme.

The association between RNase E-(628–843) and RhlB has been reported to have a *K_d_* in the region of 50 nM (19, 22). The strong binding made it possible to overexpress the RhlB·RNase E-(696–762) complex using a di-cistronic vector, and this system has proven to be a useful tool to study enzymatic activity of the WT and mutant forms of the helicase. Characterization of this protein interaction by ITC revealed the helicase/RNase E interaction to be strongly enthalpy driven, with the entropy change being highly unfavorable (Table 2). RNase E-(696 –762) is enriched in polar residues, especially acidic ones (estimated pI 5.0). Examining the RNase E sequences from other *γ*-pro-teobacteria that also encode an RhlB homologue and so can presumably form an RNase E·helicase complex, we find that the helicase binding site has a comparatively well conserved pattern of polar and non-polar residues (42). We envisage that the association of RhlB and RNase E in *E. coli* and related *γ*-proteobac- teria might involve extensive hydrogen bonding and perhaps a few new non-polar contacts, which is in accord with the polar nature of the RNase E peptide and the enthalpy-driven interaction of peptide and helicase. One way that the RNase E/RhlB interaction can be achieved is through extension of hydrogen bonding patterns from the exposed strand of the recognition partner to form a pseudo-continuous *β* sheet. There are several examples in which this mode of interaction is used in protein complexes with small cognate peptides (43). The highly polar character of the RNase E peptide may indicate that side chains also become organized through hydrogen bonding interactions upon complex formation with RhlB.

The interaction of RNase E with RhlB enhances its ATPase activity, indicating that structural changes associated with the protein/protein interaction modulate the active site. A similar enhancement in activity has been reported for the eukaryotic eIF4A DEAD box helicase upon binding to its adaptor protein eIF4G, which forms part of a larger multienzyme complex involved in translation initiation (11). NMR studies have indicated that the site of interaction for eIF4G is on the C-terminal domain of eIF4A (44). Correspondingly, the C-terminal domain of RhlB also is the likely site of interaction with RNase E.

Previous *in silico* data predicted a surface site on the C-ter- minal domain of RhlB for interaction with RNase E (23). This site contains the two residues, Ser-381 and Tyr-383, which where noted to be solvent accessible and to co-vary with the residue corresponding to His-320 in motif V among RhlB homologues. Substitution of Tyr-383 with Ala caused the protein to self-associate (Fig. 4). The mutant did not detectably bind RNase E, and did not have any observable ATPase activity in the presence of RNase E-(696–762). This mutation cannot therefore address the question of how RNase E and RhlB interact, but it does present the puzzle of why the mutation of this surface residue self-associates to occlude RNase E interactions and ATPase activity.

**Figure 3:**
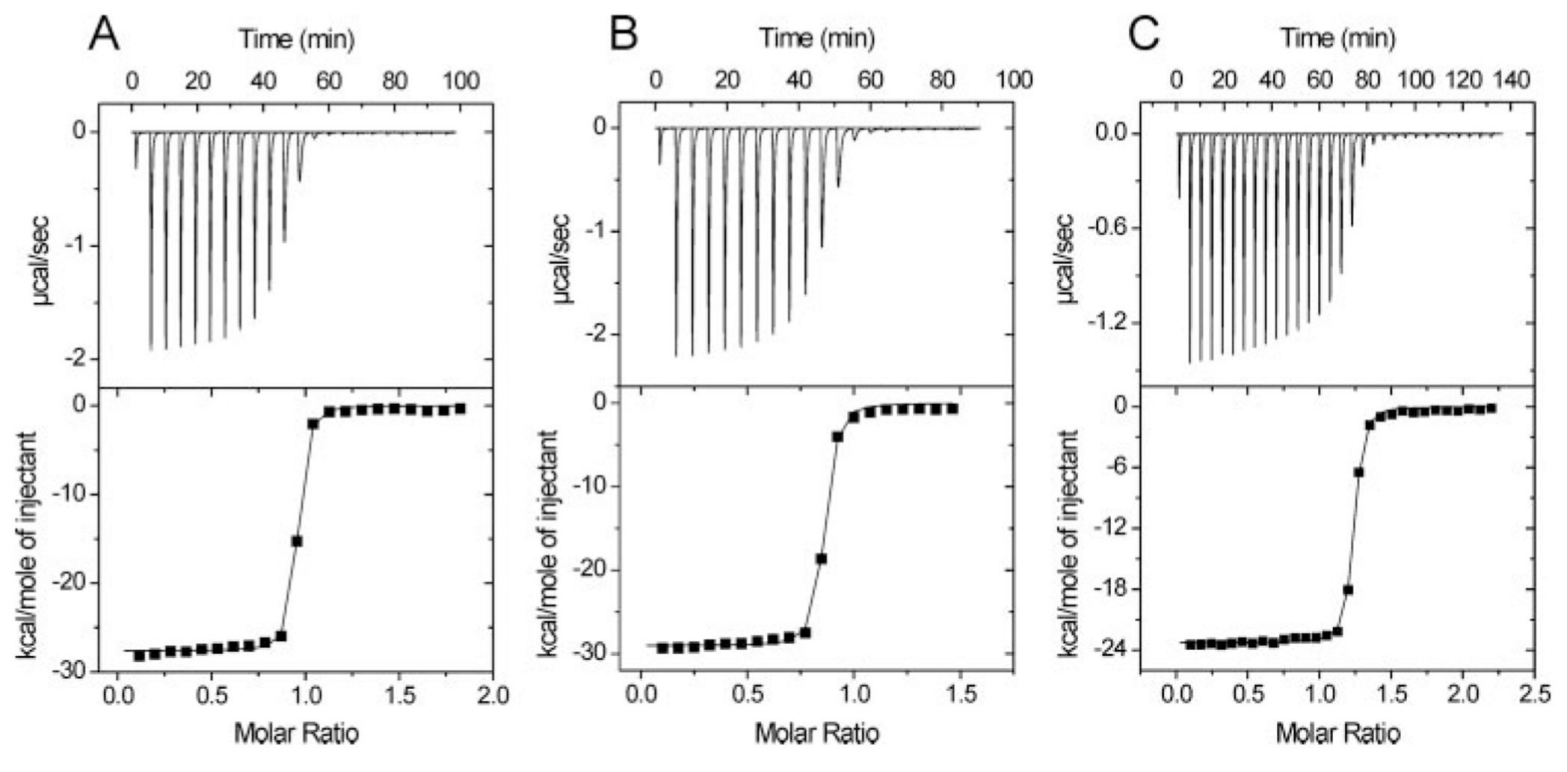
Representative ITC binding curves. Binding of RNase E-(696–762) toWT(*A*), H320D (*B*),and S381A (*C*) RhlB at 25 °C. The *top panels* show the raw data after the baseline correction and the *bottom panel* is the integrated data corrected fortheheatofdilution of RNase E-(696-762).The *solidline* in the *bottom panel* isthe best fit of the data toa 1:1 binding model with a lower limit for the *K_a_* given in Table 2.

**Figure 4:**
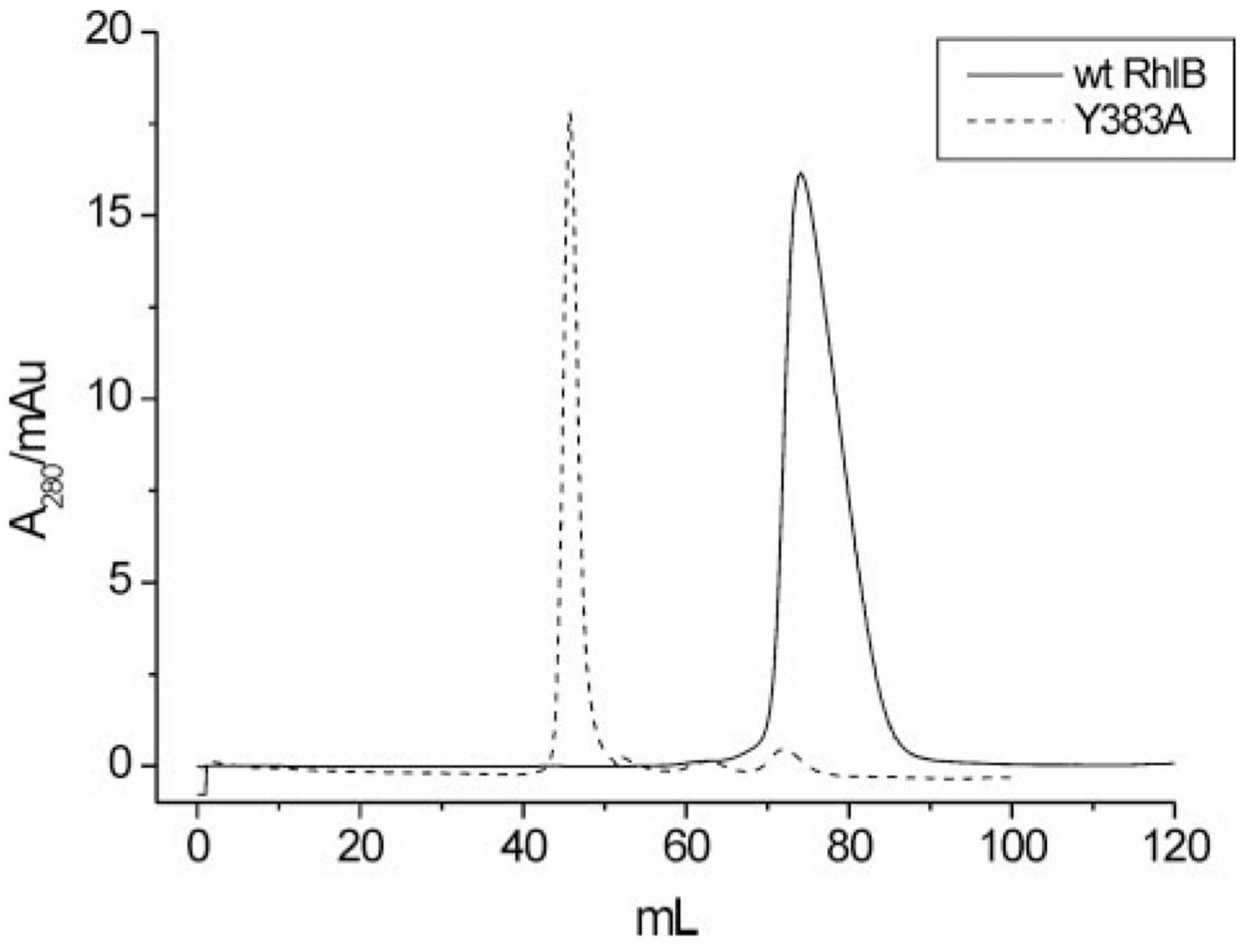
S200 size exclusion profiles for wt RhlB and the Y383A variant. The peak position corresponding to the WT protein is consistent with a monomer species, whereas the sharper peak observed for Y383A corresponds to a higher molecular weight species.

**Figure 5:**
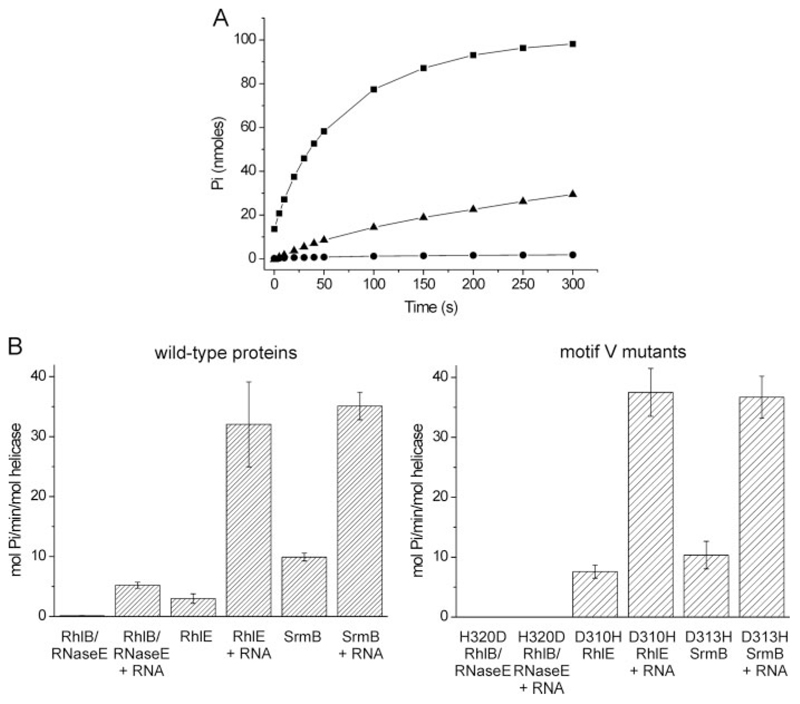
ATPase activity of WT helicases and motif V mutants. *A*, representative activity profiles for WT RhlB(·),WTRhlB/RNase E-(696–762) (Á),andWTRhlE(·) inthepresenceofbulk*S. cerevisiae* RNa.*β*,graphical representation of the ATPase activity in the absence and presence of bulk *S. cerevisiae* RNA. RNase E corresponds to RNase E-(696–762).

The data for the S381A mutant suggest, but do not prove, that this surface is involved in recognition of RNase E. We noted a smaller enthalpy component for RNase E peptide binding (Table 2 and Fig. 3). Nonetheless, the changes in enthalpy indicate that this residue affects RNase E recognition, most likely through direct or water-mediated interaction at the protein-protein interface. Ser-381 probably has a weak role in mediating communication with the active site because substitution with Ala has a negligible effect on ATPase activity (Table 3). The surface involved in RhlB/RNase E interaction is yet to be proven.

In the course of these studies, we also examined the effect of substitutions in the purine-engaging end of the nucleotide recognition pocket (Fig. 1*B*). In particular we looked at the effect of creating a potential cavity to accommodate an inhibitor with a bulky substituent by mutation of the bulky Phe-10 in physical proximity of the ATP. The residue is also in contact with residues of the Q-motif. Our data show that the RhlB mutants F10M and F10A in complex with RNase E-(696 –762) displayed almost the opposite of WT activity: namely, enhanced activity in the absence of RNA but no detectable activity in the presence of RNA (Table 4). Circular dichroism spectra confirm that the mutants are folded (data not shown). The *T_m_* for WT is roughly 43 °C, and for the F10M mutant is 48 °C.

To understand the effects of the Phe-10 mutations, it may be helpful to consider the exon-junction complex, where the ATPase activity of its DEAD box helicase component (eIF4AIII) is inhibited by interactions with RNA and the partner regulatory protein (45). The crystal structure of the exonjunction complex indicates that inactivation of the helicase likely results from small structural adjustments in its active site caused by complex formation (13, 14). Conceivably, substitution of Phe-10 in combination with binding of RNA has a similar conformational effect on the catalytic site in RhlB, or may restrict the conformational freedom of enzyme so that the active site cannot accommodate the transition state required for hydrolytic attack of the terminal phosphate.

The *in vitro* properties of the RhlB Phe-10 mutants may account for the growth arrest phenotype of yeast in which the DEAD box helicase Ded1 is mutated at the corresponding residue, namely Phe-144 (34). In the original identification of the Q-motif the isolated Phe residue was proposed to have a role in hydrophobic stacking with a conserved Pro. In RhlB and one other *E. coli* DEAD box protein, CsdA, this Pro is replaced by a Cys and Met, respectively (Fig. 1*B*). Our studies on RhlB together with the reported *in vivo* studies with Ded1, confirm that this Phe has a more important role than simply providing a hydrophobic stacking interaction.

In summary, the effects of the mutations we have studied here show that RNA binding can communicate an inhibitory structural change to the catalytic site in a Q-motif proximal mutant; and that the effects of substitutions in motif V are highly context dependent, being neutral in SrmB and RhlE but completely inactivating in RhlB. These data suggest two important aspects of RhlB structure and function. First, there exists a structural pathway of communication between the RNase E binding surface and the ATPase active site, as proposed previously. Second, they indicate that residues proximal to the Q-motif play an important role in the communication between the RNA binding surface and the ATP catalytic site.

**Figure 6:**
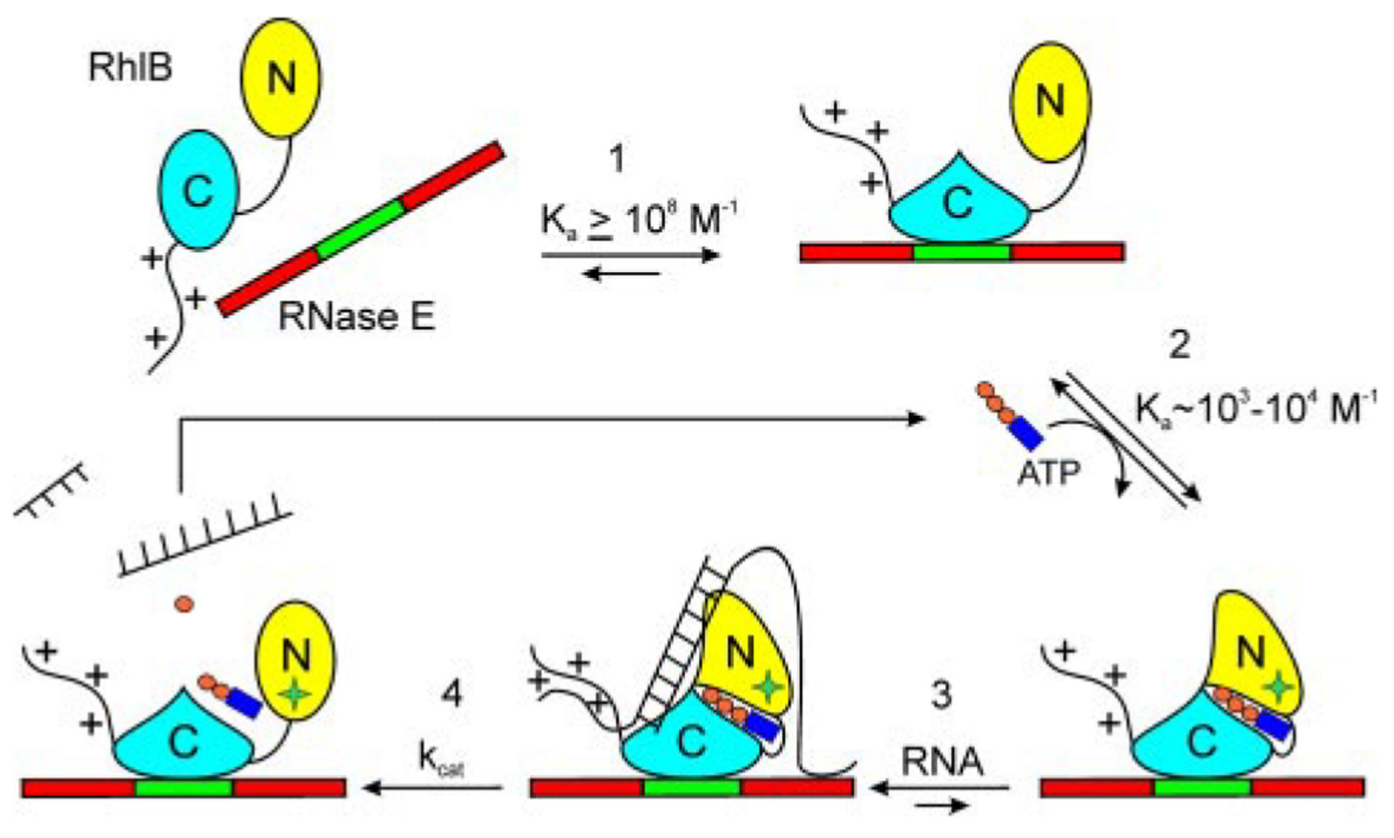
A schematic indicating a possible sequence of events in RhlB unwinding of duplex RNA. *1*) RhlB binds to a surface of RNase E encompassing residues 696-762 (*green*) causing a possible change in structure of the C-terminal domain, which transmits to the ATP binding site.*2*) The binding of ATP induces association of the N-terminal and C-terminal domains; this perhaps causes a conformational switch in the surface of the N-terminal domain that formsan additional RNA binding site. The conformational switch is communicated through the Q-motif and Phe-10. This accounts for the inhibitory effect of RNA binding on ATP hydrolysis by the Phe-10 mutants. 3) The C-ter-minal tail of RhlB and the flanking RNA-bindings sites in RNase E (*red*) both interact with RNA. Deleting the tail eliminates ATPase activity (Table 3), showing that it plays a crucial role in the cycle. 4) Hydrolysis of ATP changes the surface, causing a change in the interaction with the RNA.

Several models have been proposed for how ATP hydrolysis and helicase mechanical activities are linked. Linder and coworkers (1, 35) have proposed that the energy of ATP hydrolysis is not used to directly drive strand displacement, but to cycle between high and low affinity states for the nucleic acids. Combining this proposal and the interpretation of data presented here and in earlier reports, we present a schematic representation of the ATPase and RNA binding cycle for RhlB in Fig. 6. This illustrates how the C-terminal tail and the flanking RNA-bindings sites in RNase E both interact with RNA. Deleting the tail eliminates ATPase activity (Table 3), showing that it plays a crucial role in the cycle. ATP induces association of the N-ter-minal and C-terminal domains; this perhaps causes a conformation switch in the surface of the N-terminal domain that forms an additional RNA binding site. The conformational switch is communicated through the Q-motif and Phe-10, accounting for the inhibitory effect of RNA binding on ATP hydrolysis by the Phe-10 mutants. Finally, hydrolysis of ATP is likely to change the surface, causing change in the interaction with the RNA.

It seems that the properties of the RhlB helicase are dependent on subtle aspects of its fold, a point that is also emphasized by the context dependent effects for mutations in motif V noted here. This point is further highlighted by the implication that the ATPase active site can be modulated from a surface that is distal to both the site and the RNA binding surface, and from the effect of Q-motif proximal mutation. Judging from the inhibitory effects of RNA in the Phe-10 mutants, it seems that there must be a finely tuned balance between the strength of binding of RNA substrate and the efficiency of harvesting the potential work of ATP hydrolysis. We envisage that not only RhlB, but other DEAD box RNA helicases generally may have extensive internal networks of side chain interactions that are linked to affect conformational adjustments required for activation, RNA interaction, and the finely adjusted gearing for transduction of chemical energy into mechanical work.

## Figures and Tables

**Table 1:** Mutagenic primer pairs used to generate the various mutations in the recombinant RNA helicases In lower case and underlined are the nucleotides that are changed from the original sequence to generate the respective helicase mutation.

Mutation	Mutagenic primer pairs
**RhlB**	
F10A	5*’*-CAGAACAGAAGgçTTCCGACTTCGCCC-3*’*
	5*’* -GGGCGAAGTCGGAAgçCTTCTGTTCTG-3’
F10M	5’-CAGAACAGAAGaTgTCCGACTTCGCCC-3’
	5’ -GGGCGAAGTCGGAcAtCTTCTGTTCTG-3’
H320D	5’-GCCGCGCGTGGTTTGUgATATTCCGGCAGTGACG-3’
	5’ -GCGTCACTGCCGGAATATcCAAACCACGCGCGG-3 ‘
S381A	5’-GGTCACTCAATTCCGGTAgçCAAATACAATCCGGACG-3’
	5’-CGTCCGGATTGTATTTGgçTACCGGAATTGAGTGACC-3’
Y383A	5’-CCGGTAAGCAAAgçCAATCCGGACGCATTG-3’
	5’-CAATGCGTCCGGATTGgçTTTGCTTACCGG-3’
**RhlE**	
D310H	5’-CGCTGCGCGCGGCCTGçATATTGAAGAGCTGC-3’
	5’ -GCAGCTCTTGAATATgCAGGCCGCGCGCAGCG-3’
**SrmB**	
D313H	5*’* -GCCGCGCGCGGTATCçACATTCCTGACGTCAG-3’
	5’-CTGACGTCAGGAATGTgGATACCGCGCGCGGC-3’

**Table 2:** Thermodynamic parameters of RNase E-(696-762) binding to RhlB and site-directed variants obtained from ITC Experiments were performed at 25 °C in 100 mM KP_i_, pH 7.4, 50 mM NaCl, 1 mM dithiothreitol. The units of Δ*G*_B_, Δ*H*_B_, and *T*Δ*S*_B_ are kcal.mol^-1^.

RhlB	N^*a*^	*K_a_^b^*	Δ*G*_B_	Δ*H*_B_	*-TδS*
WT (3)	0.92 ± 0.02	1.1 ± 0.2	-11.0 ± 0.1	-27.6 ± 0.1	16.6 ± 0.1
H320D (3)	0.83 ± 0.01	0.7 ± 0.1	-10.7 ± 0.2	-29.0 ± 0.1	18.3 ± 0.2
S381A (2)	1.2 ± 0.01	0.8 ± 0.1	-10.8 ± 0.2	-23.3 ± 0.1	12.5 ± 0.1
Y383A (2)	NB^*c*^	NB	NB	NB	NB

^*a*^ Stoichiometry of binding. ^*b*^ Equilibrium association constant, 10^8^ m^-1^. Because the binding event is very strong, the association constants representlower limits; therefore, the free energy change and entropy changes also represent lower limits. ^*c*^ No binding heat change detected. The parameters were obtained by profile fitting of the individual isotherms. The number of experiments for WT RhlB and each variant is given in parentheses with the errors being the mean ± S.D.

**Table 3:** ATPase activities ofthree *E. coli* DEAD box proteins, and site-directed variants in the presence and absence of RNA and RNase E-(696–762)

Protein	Activity without RNA*a*	Activity with RNA*a*
WT RhlB	≤0.5^*b*^	≤0.5^*b*^
WT RhlE	2.9 (0.8)	32.5 (7.1)
WT SrmB	9.3 (0.7)	34.1(2.3)
H320D RhlB	ND^*c*^	ND^*c*^
D310H RhlE	7.6 (1.1)	37.5 (4.0)
D313H SrmB	10.4 (2.3)	36.1 (3.5)
WT RhlB/RNase E-(696–762)^*d*^	≤0.5^*b*^	5.2 (0.5)
RhlBΔ1–397/RNase E-(696–762)^*d*^	≤0.5^*b*^	≤0.5^*b*^
H320D RhlB/RNase E-(696–762)^*d*^	ND^*c*^	ND^*c*^
S381A RhlB	≤0.5^*b*^	≤0.5^*b*^
S381A RhlB + RNase E-(696–762)^*d*^	≤0.5^*b*^	9.4 (2.2)
Y383A RhlB	ND^*c*^	ND^*c*^
Y383A RhlB + RNase E-(696–762)^*e*^	ND^*c*^	ND^*c*^

^*a*^ The activities are expressed in moles of inorganic phosphate released per minute per mol of protein (mol/Pi/min/mol protein). The errors given in parentheses are the mean ± S.D. ^*b*^ An upper estimate of activity for proteins that display activity on theborderline for the sensitivity of the assay. ^*c*^ Activity not detectable after 10 min. ^*d*^ Co-expressed. ^*e*^ Added stoichiometrically.

**Table 4:** ATPase activity of the co-expressed Phe-10 variants of RhlB in complex with RNase E-(696–762)

Protein	Activity without RNA^*a*^	Activity with RNA^*a*^
F10A/RNase E-(696–762)	1.1 (0.6)	ND^*b*^
F10M/RNase E-(696–762)	5.4(0.7)	ND

^*a*^ The activities are expressed in moles of inorganic phosphate released per minute per mol of protein (mol/Pi/min/mol protein). The errors given in parentheses are the mean ± S.D. ^*b*^ Activity not detectable after 10 min.

## Footnotes

4 J. A. R. Worrall and B. F. Luisi, unpublished results.

5 The abbreviations used are: WT, wild type; ADP-PNP, 5’-adenylyl-*β*,*γ*-imido-diphosphate; ITC, isothermal titration calorimetry; Ni-NTA, nickel-nitrilotriacetic acid.
